# FISH Oracle: a web server for flexible visualization of DNA copy number data in a genomic context

**DOI:** 10.1186/2043-9113-1-20

**Published:** 2011-07-28

**Authors:** Malte Mader, Ronald Simon, Sascha Steinbiss, Stefan Kurtz

**Affiliations:** 1 Department of Pathology, University Medical Center Hamburg-Eppendorf, Martinistrasse 52, 20246 Hamburg, Germany; 2 Center for Bioinformatics, University of Hamburg, Bundesstrasse 43, 20146 Hamburg, Germany

## Abstract

**Background:**

The rapidly growing amount of array CGH data requires improved visualization software supporting the process of identifying candidate cancer genes. Optimally, such software should work across multiple microarray platforms, should be able to cope with data from different sources and should be easy to operate.

**Results:**

We have developed a web-based software *FISH Oracle *to visualize data from multiple array CGH experiments in a genomic context. Its fast visualization engine and advanced web and database technology supports highly interactive use. FISH Oracle comes with a convenient data import mechanism, powerful search options for genomic elements (e.g. gene names or karyobands), quick navigation and zooming into interesting regions, and mechanisms to export the visualization into different high quality formats. These features make the software especially suitable for the needs of life scientists.

**Conclusions:**

FISH Oracle offers a fast and easy to use visualization tool for array CGH and SNP array data. It allows for the identification of genomic regions representing minimal common changes based on data from one or more experiments. FISH Oracle will be instrumental to identify candidate onco and tumor suppressor genes based on the frequency and genomic position of DNA copy number changes. The FISH Oracle application and an installed demo web server are available at http://www.zbh.uni-hamburg.de/fishoracle.

## Background

In the recent years, high resolution genomic tiling arrays and SNP chips have become the standard technology to analyze copy number variations in cancer genomes. Modern arrays are inexpensive and allow for determining copy number changes at the resolution of individual genes. Gains or deletions of chromosomal material are often highly variable in size, ranging from several kilobases to entire chromosomes. One important strategy to reveal genetic loci containing putative cancer genes is to perform multiple experiments and identify chromosomal regions representing minimal common alterations. Since large alterations spanning many megabases are typically more common than the small ones containing only a few genes, as many experiments as possible should be included into such kind of analysis. Public databases like the *Stanford Microarray Database *[1], *ArrayExpress *[2], the *caArray Data Portal *[3], the *Cancer Genome Project *[4] or the *Gene Expression Omnibus *(GEO) [5], provide an unprecedented source for genomic copy number data, which may be combined with own data for a meta-analysis. In the following we will use the term *array CGH *(*array comparative genomic hybridization*) as a synonym for methods generating copy number data including classical array CGH tiling microarrays or SNP microarrays. Although a number of software tools for array CGH analysis and visualization are available — both from academia and commercial vendors — they are often limited to a particular data format, cannot be easily operated, or lack interactivity.

**Figure 1:**
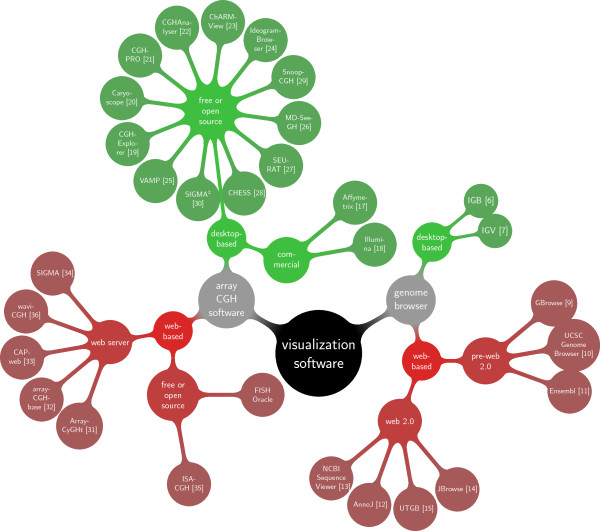
**Classification tree of software tools for array CGH data analysis**. At the root level (black), tools are grouped into general genome browsers and software specific for array CGH data analysis (gray). At the next level, we distinguish web-based software (red) from desktop-based software (green). The lowest level divides free or open source software from commercial or closed-source applications or, in the case of web-based genome browsers, pre-web 2.0 from web 2.0 applications.

Existing software tools for the visualization of array CGH data can be grouped into different ways, i.e. according to their application type (generic genome browser or pure array CGH analysis) or according to their architecture as a desktop or a web-based application (Figure 1).

The *Integrated Genome Browser *(IGB) [6] and *Integrative Genomics Viewer *(IGV) [7] are general desktop-based genome browsers. The IGB software is based on GenoViz [8], a software library for genome visualization. IGB is an open-source software allowing to display gene structure annotations, genomic alignments of expression array target sequences and EST/cDNA genomic alignments. The different kinds of data loaded from a data source are shown in different sortable horizontal tracks. IGV is an open source desktop-based tool for displaying various types of data including copy number variation data, loss of heterozygosity data, gene expression data, significant DNA aberrations, sequence alignments, and mutations. These data can be displayed using four different types of graphs, namely heatmaps, bar charts, scatter plots, and line plots.

By now, a variety of generic web-based genome browsers have been developed. Some, such as *GBrowse *[9], the *UCSC Genome Browser *[10] or the *Ensembl Genome browser *[11], are classical server-centered web-based applications, fetching data and calculating images for a specific chromosomal region before embedding it into a static web page and sending it to the client. One disadvantage of this technique is the large amount of data traffic required for creating and transferring images of genomic regions with dense information content.

In contrast, recent browsers like *AnnoJ *[12], the *NCBI Sequence Viewer *[13], *JBrowse *[14], or the *University of Tokyo Genome Browser *(UTGB) [15] are *Rich Internet Applications *(RIAs) based on a technology called *asynchronous JavaScript and XML *(AJAX) [16]. This allows rendering images at the client side as well as loading server side data dynamically without having to refresh the whole page, thus reducing both the required data traffic and the server load.

All web-based browsers share the property of being generic in nature. Although they provide many extensions, it is sometimes not possible or at least difficult to achieve the desired visualization. For this reason, several specialized software tools for processing and visualizing array CGH data have been developed. The Affymetrix *Genotyping Console *[17] and the Illumina *GenomeStudio Software *[18] are commercial desktop-based software products, capable of handling different microarray data, including array CGH data. Their main disadvantage is that they are both limited to the respective vendor-specific array platform.

In academia, several open source or freely available desktop applications specific for array CGH data have been developed, including *CGH-Explorer *[19], *Caryoscope *[20], *CGHPRO *[21], *CGHAnalyzer *[22], *ChARMView *[23], *IdeogramBrowser *[24], *VAMP *[25], *MD-SeeGH *[26], *SEURAT *[27], *CHESS *[28], *SnoopCGH *[29] and *SIGMA2 *[30], written in Java or C++. With the exception of CGHAnalyzer, all offer an interactive display of array CGH and/or gene expression data. Their support of additional features varies extensively (see Tables S1 and S2 in the additional file 1). The main disadvantage of these tools is that each installation of a program needs to be run on a separate computer, requiring additional effort to keep the software and data up-to-date across release updates. Thus they are not well suited for a distributed, collaborative approach to genome research.

Finally, the group of web-based software for visualization of array CGH data comprises *ArrayCyGHt *[31], *arrayCGHbase *[32], *CAPweb *[33], *SIGMA *[34], *ISACGH *[35] and *WaviCGH *[36]. All of these are primarily accessible via static installations on web servers, requiring to upload the data to be analyzed to external parties. While this supports collaboration, it may raise problems related to privacy concerns or a large volume of necessary data which could become a heavy burden for the server.

Table S3 in the additional file 1 lists the different features of existing web-based software tools for visualizing and analyzing array CGH data. Interestingly, except for waviCGH, all web-based software tools for array CGH data analysis have been published in the mid-2000s. However, waviCGH, published in 2010 and focused on automatic analysis and visualization of array CGH data in a genomic context, does not provide a dynamic visualization. Instead it produces static, chromosome-wide images of the data.

We have developed a software tool called *FISH Oracle *combining the most important features of the above mentioned software tools for visualizing array CGH data:

First of all, FISH Oracle does not impose a limit on the number of array CGH experiments to be visualized at once. This is important since a large number of experiments is often necessary to obtain accurate results, a fact confirmed by the large number of available data. Secondly, FISH Oracle provides the relevant genomic context, i.e. besides the segment data it displays annotations available in Ensembl [37] at a genomic resolution ranging from ten to 10 million base pairs. This feature is important because the task of identifying new chromosomal aberrations and single genes overlapping with copy number variations requires observation of the relevant data on different scales. Detailed information about a single gene or other functional elements (e.g. their UniProt [38] identifier) can be obtained in FISH Oracle by clicking on the corresponding element. This feature is important as users quickly want to decide whether the functional element in question could be a possible target for further investigation.

FISH Oracle stores its data in a central database. Once uploaded, it can quickly be accessed for any user of the system, thus reducing data redundancy (compared to desktop applications) and allowing collaborative work based on the data. The fast visualization engine in combination with advanced web and database technology supports highly interactive use. FISH Oracle comes with a convenient data import mechanism, powerful search options for genomic elements (e.g. gene names or karyobands) and mechanisms to export the visualization into different high quality formats.

We termed our software FISH Oracle because it is well suited for computational selection of candidate genes for subsequent *fluorescence in situ hybridization (FISH) *experiments.

We tested the application using two different data sets. One data set consists of SNP microarrays. It includes our own data, data from the Sanger Cancer Genome project [4] as well as data from NCBI GEO. The other data set comprises two channel microarray data from NCBI GEO.

## Results and Discussion

### User interface

The data import process in FISH Oracle consists of two steps. In a first step, the data are uploaded to the server in form of a tab-delimited file. Each line in the uploaded file specifies a segment by an identifier for the chromosome it comes from, its start position, its end position, its mean intensity value and its number of markers. In the second step, the user specifies a study name, the tissue type and the microarray type for the uploaded data. Further information about the pathological state, as well as a detailed description of the data source, can optionally be added. Once the annotation file is uploaded, the data are checked for consistency and stored in a relational database.

**Figure 2:**
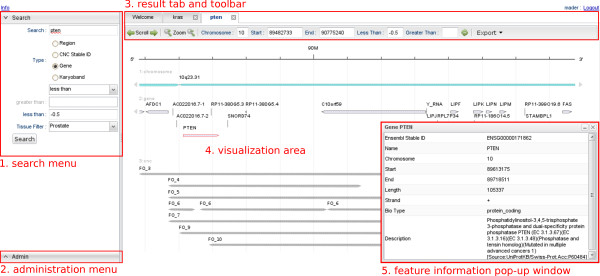
**The FISH Oracle user interface**. (1) The search menu is on the left hand side. It allows to specify a region, segment IDs, gene names and karyobands as search keys. If the user wants to display a certain genomic region, the search box is replaced by three text fields to enter the chromosome and start and end positions. A threshold for the segment mean intensity values can be specified with the "less than" or "greater than" option. Typically, the "less than" option is used with a negative threshold to focus the visualization to segments with negative mean intensity value (deletions). The "greater than" option is used with a positive threshold to focus the visualization to segments with positive mean intensity value (amplifications). The tissue type filter restricts the display to segments for one ore more specific tissue types. (2) The administration menu (lower left corner) provides functionality for data import and user account administration, e.g. activation of recently registered users. (3) Each search opens a new tab that can be identified by the search query appearing as the caption of the tab. Each open tab has its own toolbar, showing the exact location of the displayed region as well as the current thresholds. The toolbar provides buttons for zooming into the displayed region, scrolling along a chromosome, and exporting the displayed image for download to the user's computer. (4) The visualization, according to the current toolbar settings, is displayed below the toolbar. In this case, the image shows segment data and annotations in the region of the gene *PTEN*. (5) Clicking on the symbol representing a gene or a segment triggers a pop-up window containing corresponding detailed information.

Once the segment data are stored in the database and the corresponding annotation is available, the user decides which region of the considered genome is to be displayed. This can be done by specifying one of the following location markers: range of genomic positions, name of a gene or karyoband, or segment ID (Figure 2). FISH Oracle then displays the genome annotation and segment data at the specified genomic location or in the region containing the specified item. The initially displayed genomic region depends on the extent of found segments. If the search term is a gene or a karyoband and no segments are found, the displayed range is equal to the length of the karyoband or the visualized range is extended by 200% of the gene size in both chromosomal directions. If only one segment is found by a segment search, the displayed range equals the segment size. The maximum initial range is 20 Mbp.

Segments are selected according to a user-specified threshold for the mean intensity values. This threshold can be specified in two modes: In the "less than" mode, all segments whose mean intensity value is less than the threshold are displayed, allowing to select segments representing deletions. Similarly, the "greater than" mode selects segments with a mean intensity value larger than the threshold. Thus this mode allows to select segments representing amplifications. In addition to the threshold, a combo box allows the user to restrict the selection to segments that originate from experiments for specific tissues.

Each search delivers an image with up to three tracks. A track possibly consists of several lines if elements of a track or their captions overlap. This makes them more readable. The *karyoband track *is always shown and it appears as the top track. The *gene track *shows, for the specified region, all genes according to the Ensembl annotation of the genome. The *segment track *shows, for the specified region, all segments according to the currently chosen thresholds and tissue types. At the top of the image a genomic scale depicts the shown region of the chromosome. A toolbar shows the exact chromosomal coordinates of the displayed image and contains control buttons for scrolling over the chromosome and zooming into or out of the chromosome. Clicking on the parts of the image representing genes or segments delivers a pop-up window showing additional information on the corresponding element (Figure 2).

FISH Oracle also allows for export of the shown data (segments and annotation) in a tabular representation to a file in Microsoft Excel format.

The visualization of the segments and annotation can be exported as PNG bitmaps or in the PDF, PostScript, or SVG vector graphics format.

### Application of FISH Oracle to our own dataset

In a first study, we applied FISH Oracle to our own array CGH data sets (231 experiments) which were obtained from experiments using different human cancer cells and Affymetrix SNP 6.0 microarrays. We also used parts of the Sanger Cancer Genome Project (CGP) [4] (Affymetrix SNP 6.0 microarrays, 5 experiments) and NCBI GEO [39,40] (Affymetrix Mapping 250K Nsp SNP microarrays, 9 experiments) which were randomly selected. We will refer to this data set as *FISH Oracle data*.

All array CGH data sets (given as CEL files) were normalized based on an internal reference. This means that every intensity value of a specific probe set is divided by the mean intensity value over the 0.25- and 0.75-quantile of the same probe set of different microarrays. This allows normalization of the data without reference arrays. We applied DNAcopy [41] to the normalized data to calculate breakpoints of intensity values. The result is a tab-delimited file with segments characterized by consecutive positions of similar intensity values. Each segment is associated with a chromosome number, a start and end position on the chromosome, the number of SNP markers covered by the segment and the mean intensity value of all SNP markers contained in the segment. All resulting tab-delimited files were uploaded to FISH Oracle.

**Figure 3:**
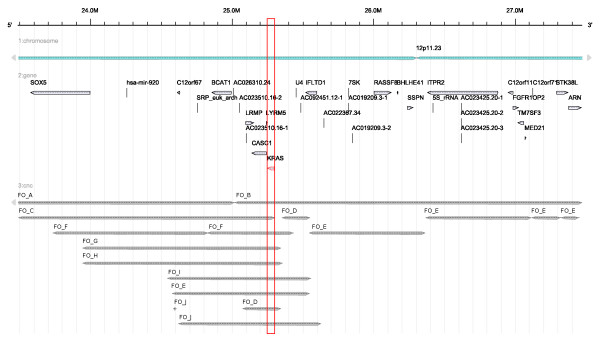
**Annotated genes and segment data obtained from different array CGH experiments for esophagus, pancreas, prostate, colon and multiple myeloma tumor tissue shown at the 12p11.23 locus**. The segment data are taken from the FISH Oracle data. All segments have a mean intensity value greater than 0.5 and therefore represent amplified regions. There are amplifications in the region from about 24.5 Mbp to 25.6 Mbp. The minimal overlapping region covers the area from 25.1 Mbp to 25.35 Mbp. This region contains the genes *LRMP*, *CASC1*, *LYRM5 *and *KRAS*. *KRAS *is a known proto oncogene, which may lead to tumor development if amplified [42]. The minimal region including *KRAS *is shown in a red box.

**Figure 4:**
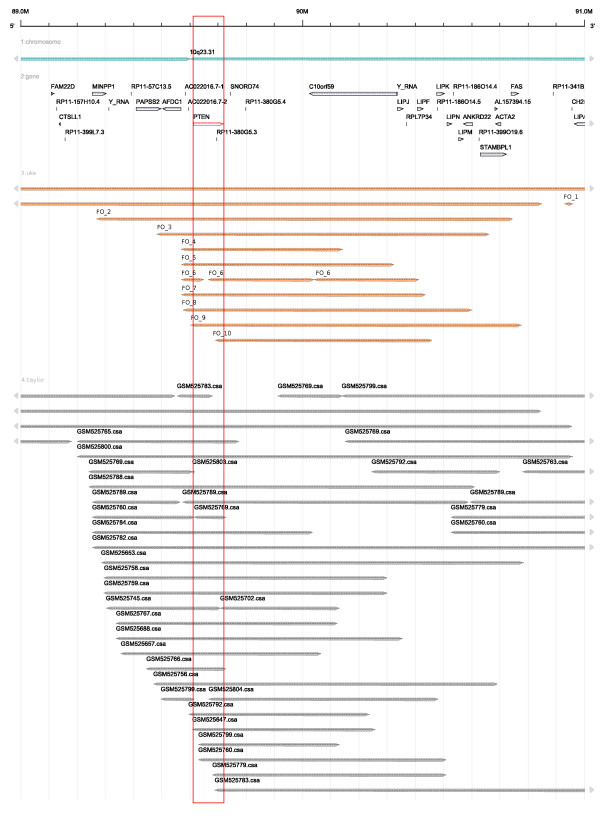
**Annotated genes and segment data obtained from different array CGH experiments for prostate tumor tissue shown at the 10q23 locus**. The segments colored in orange correspond to the FISH Oracle data. The gray segments are derived from the Taylor data. Both data sets show an accumulation of segments in the region from 89 Mbp to 91 Mbp. The minimal overlapping region indicated by the FISH Oracle data extends from about 89.6 Mbp to 90.2 Mbp, containing the genes *PTEN *and *C10orf59*. The minimal overlapping region indicated by the Taylor data overlaps almost exactly with the gene *PTEN*. The region around *PTEN *is shown in a red box. It overlaps with several deletions, as only segments with a mean intensity threshold less than -0.7 for the Taylor data and less than -0.35 for the FISH Oracle data, are shown. *PTEN *[43] is a known tumor suppressor gene which can lead to tumor development if it is deleted. Especially in prostate tissue, the deletion of a chromosomal region containing *PTEN *leads to tumor development.

**Figure 5:**
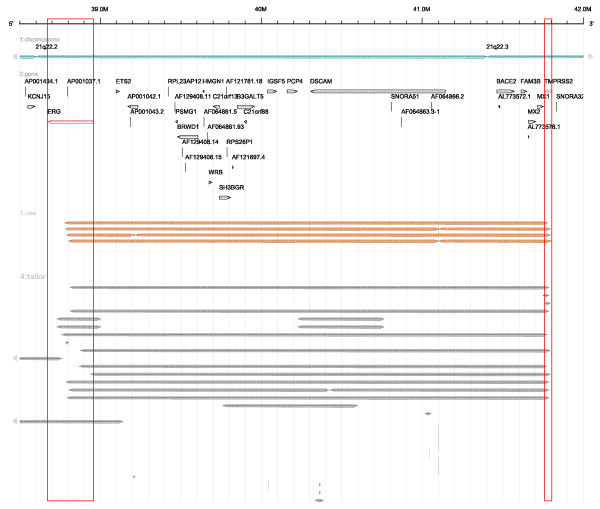
**Annotated genes and segment data obtained from different array CGH experiments for prostate tumor tissue shown at the 21q22 locus**. The segments below a threshold of -0.55 for the Taylor data (colored gray) and -0.25 for the FISH Oracle data (shown in orange) indicate interstitial deletions covering a 3 Mbp genomic segment. The breakpoints are located within the genes *TMPRSS2* and *ERG* (indicated by red boxes), leading to the characteristic *TMPRSS2:ERG* fusion bond in about 40-60% of prostate cancers [44]. Segment captions are not shown to make the resulting image more compact.

We show by three examples how the interactive visualization provided by FISH Oracle reveals coincidences between an accumulation of segments from different experiments on one side and annotated genes in a specific region on the other side. The first region (Figure 3) is located around the 12p locus of the human genome with several amplifications overlapping the *KRAS *gene. The second region (Figure 4) can be found around the 10q23 locus with several deletions overlapping the *PTEN *gene. The third region (Figure 5) is located around the 21q22.2/21q22.3 loci with several deletions overlapping the genes *TMPRSS2 *and *ERG*. These coincidences are consistent with previous publications on the relevance of these genes for cancer: *KRAS *is a proto oncogene [42], *PTEN *is a tumor suppressor gene playing an important role in prostate cancer [43] and *TMPRSS2:ERG *is a known fusion gene in prostate cancer [44]. The three examples show that FISH Oracle has the potential to aid a researcher in deriving interesting new hypotheses about potential cancer genes based on segment data and corresponding annotations.

### Application of FISH Oracle to foreign data

The second study is based on the data of Taylor et al. [45] who analyzed 231 prostate carcinomas using different types of microarrays, mainly 244K Agilent human array CGH microarrays. The data are available as text files from NCBI GEO (accession number: GSE21035). We will refer to this data set as *Taylor data*. As the Taylor data is based on two color microarrays (including, for each patient, one tumor tissue sample and one healthy tissue sample as reference) we had to use another normalization method. The data were normalized based on global medians using the method *normalizeWithinArrays *from the R package *limma *[46]. Segment data were calculated using DNACopy.

Figure 4 and Figure 5 confirm that the Taylor data are consistent with the FISH Oracle data. As the Taylor data originate from considerably more experiments than the FISH Oracle data, the former more clearly reveals important locations with deletions (like the location near 10q23 or 21q22.2/21q22.3).

### Thresholds for segment mean values

**Figure 6:**
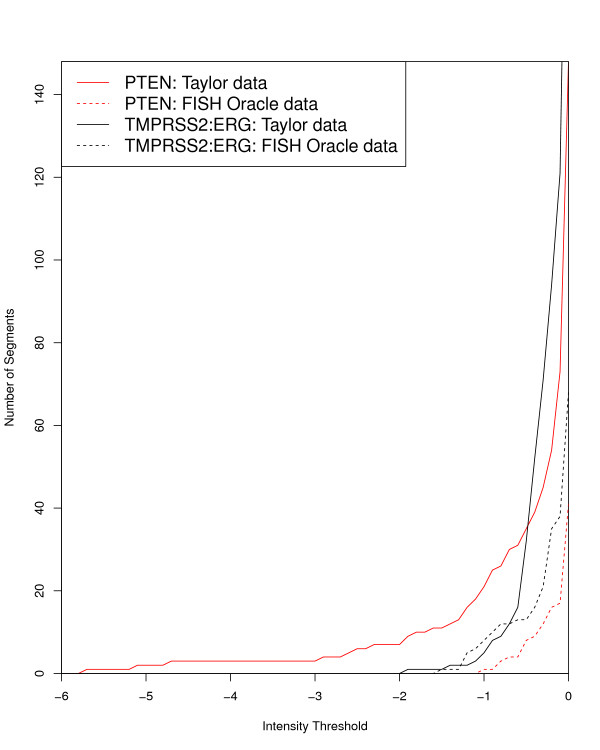
**Segment counts (Y-axes) as a function of the threshold for the mean intensity value (X-axis)**. The solid lines are based on the Taylor data and the dashed lines are based on the FISH Oracle data. The red colored lines refer to segments in the minimal chromosomal region containing the gene *PTEN *(see also Figure 4) while the black colored lines refer to segments in the region 21q22.2/21q22.3 (see also Figure 5).

Intensity values for segments originate from the logarithmic transformation of sample to reference sample ratio and can be positive or negative [47]. A user-specified mean intensity threshold determines which segments are displayed. A negative threshold selects segments with a (negative) mean intensity value not larger than the threshold. These segments represent deleted genomic regions. A positive thresholds selects segments with a (positive) mean intensity value not smaller than the given threshold. These segments represent amplified genomic regions. A reasonable threshold may be adjusted experimentally. This is exemplified in Figure 6 showing the distribution of the number of segments depending on the threshold. The counts refer to the FISH Oracle data and the Taylor data, focusing on the regions 10q23 and 21q22.2/21q22.3 already considered in Figures 4 and 5. The short response time of the FISH Oracle visualization allows to quickly explore the effect of different thresholds.

The comparison of the FISH Oracle data with the Taylor data reveals the influence of data quantity: The segment counts derived from the FISH Oracle data are approaching zero much faster than the segment counts derived from the Taylor data. Hence in the FISH Oracle data it is more difficult to spot regions with significant amplifications or deletions. This problem also became obvious in the visualization of the FISH Oracle data at the 21q22.2/21q22.3 loci where the significant segments could hardly be distinguished from noise. In contrast, the larger Taylor data set shows a much more accurate picture of interesting regions.

### Discussion

We have developed FISH Oracle, an interactive web-based application to visualize segment data from an unlimited number of array CGH experiments in the context of gene annotations. Functional elements and segments are presented in a clear and concise fashion. Moreover, the zooming capability of the system makes it possible to display all elements at the resolution desired by the user. Easy to use filters allow to select groups of segments to be visualized. We expect that the high quality of the visualization and the flexibility of the software will enable life scientists to quickly derive interesting hypotheses about candidate cancer genes occurring in amplified or deleted regions. To communicate their findings, users can quickly export the generated images in different high quality formats, e.g. for publication or post-processing using standard graphics software. FISH Oracle is flexible regarding the underlying genome as long as the segment data refer to the same sequence basis as an annotation data set that is available in Ensembl. For example, segment data sets from the mouse can be used with FISH Oracle.

Even though the images in FISH Oracle are generated at the server side of the application, only the image itself is retransmitted and replaced at the client side. Additional gene annotation information for a specific gene is loaded from the database when it is needed. In a "classical" server centered web application all additional gene annotation information would have to be loaded concurrently with the visualization of the data, significantly increasing the data transfer rates in particular when visualizing regions with high gene density.

While many of the features of FISH Oracle are available in general genome purpose browsers, they are not always available in the software tools specific for array CGH data.

In contrast to most other web-based applications for visualizing array CGH data, FISH Oracle is able to visualize an unlimited number of segments in the chosen chromosomal region at low and high resolution. Most desktop-based applications also provide the visualization of multiple segments. However, with an increasing number of segments the resulting visualizations of the desktop tools become more dense, making it more difficult for the user to maintain an overview. In other cases, desktop-based software does not provide a high resolution view of all segments, complicating the search for single genes overlapping with copy number changes.

FISH Oracle stores the imported data persistently in a database. In contrast, the desktop-based array CGH software solutions load the data from text files and store them in internal data structures. Thus in each session the input must be re-imported. For large data sets involving mandatory preprocessing or manual loading of several data sets (e.g. SnoopCGH) the import becomes cumbersome for the user.

The web-based applications for processing array CGH data (see introduction) are mainly offered as publicly available web servers. Additionally CAPweb and arrayCGHbase can be obtained for local installation by requesting it from the maintainers. FISH Oracle is available as a web server and additionally as an open source package at http://www.zbh.uni-hamburg.de/fishoracle. We have made some effort to keep the installation as easy as possible.

To the best of our knowledge there is no single tool for processing array CGH data offering a comparable visualization functionality (see Tables S1-3 in the additional file 1). In each of the desktop-based software tools, at least one core functionality is missing when comparing it to FISH Oracle. Most of the desktop-based tools do not provide a visualization of the genomic context, do not support alternative genomes, and do not provide high-quality image export. Not all of them offer built-in normalization or segmentation procedures. For some of them, the license conditions are not specified. MD-SeeGH [26] is probably the desktop-based software that comes closest to FISH Oracle in terms of visualization capabilities. (see Table S2 in the additional file 1). However, MD-SeeGH is only available for MS-Windows. Other tools, such as CHESS [28], are apparently unavailable.

Several of the web-based tools do not provide interactive visualization or genome browsing capabilities (see Table S3 in the additional file 1). Often the web-based tools are specifically tailored to a fixed set of genomes, or (as in the case of SIGMA [34]) are restricted to a specific database and do not provide interfaces to common data formats. The ISACGH software [35] is no longer available on its own. Neither is the GEPAS toolkit it is built upon, and which has been merged into the Babelomics software suite [48]. The ISACGH software also lacks integrated genome browsing functionality, and instead provides hyperlinks to Ensembl.

ArrayCGHbase with its "chromosome view" is the web-based software that comes closest to FISH Oracle. While both software-tools have similar capabilities regarding the visualization of segments data, they differ in the kinds of additional data displayed: FISH Oracle focuses on additional gene annotations which are not handled by arrayCGHbase. On the other hand, arrayCGHbase allows the display of raw intensity values which is not displayed by FISH Oracle. Considering the use of both tools, it becomes apparent that FISH Oracle pursues a different approach to data visualization than arrayCGHbase. ArrayCGHbase is centered on experiments, coming with filters to select certain experiments, whose data can be visualized using different methods. In contrast, FISH Oracle is centered on genome annotations. Once logged into the application the user can immediately search for regions, karyobands or genes of interest.

In summary, both tools are unique in their own way and complement each other well.

While FISH Oracle does not contain explicit segmentation, normalization or quality assessment components, its open input format allows researchers to combine various specialized tools for these tasks with the visualization capabilities of FISH Oracle. This option makes the software particularly attractive to life scientists analyzing array CGH data.

On the client side, all software that is needed to access FISH Oracle is a recent web browser with JavaScript support enabled. On the server side, the software requirements are more extensive (see Methods section). With regard to hardware requirements, it is possible to install the FISH Oracle server software on a standard Linux workstation with at least 1 GB RAM. The hard disk space requirement is largely dominated by the size of the genome annotations. For example, a mirror of the human genome annotation data from Ensembl requires about 14 GB of hard disk space.

## Conclusions

Our examples show that FISH Oracle is a powerful tool to detect amplifications and deletions of chromosomal regions containing proto oncogenes, tumor suppressor genes and fusion genes. Comprehensive search options, the dynamic visualization of multiple microarray experiments and export of high quality images are useful functions to cope with todays amounts of data. State of the art web and database technology facilitate collaborative work. Altogether FISH Oracle represents a helpful tool for life scientists in the search of potential candidate cancer genes.

## Methods

### Data storage

FISH Oracle uses the MySQL relational database to store its source data. In particular, two different kinds of data are stored in two separate databases: genome annotation data (as available in the Ensembl database [37]) and segmented array CGH data. The segment data are parsed from text files uploaded to the web-server. Access to the Ensembl database is established by the EnsJ Java library [49]. The connection to the desired target database can be configured by the administrator. For example, it is possible to obtain the annotation information from a remote database (accessed via the Internet) and the segment data from a database server in a local network. Splitting the data into two databases has the advantage that the data sources for the gene annotation can easily be switched or updated without the need to change the database storing the segment data, and vice versa.

### User interface and server service

The user interface of FISH Oracle is written in the Java programming language. This includes both the client side of the web application (running in the user's web browser) and the server side (running on the web server). To reliably integrate both sides, we make use of the *Google Web Toolkit *(GWT) [50]. The GWT programming framework compiles a unified application code written in Java into both JavaScript (for client-side use) and Java servlet bytecode (for use on the server side). It implements a convenient and efficient mechanism for client-server communication. Also, the resulting web applications are compatible with all common web browsers. Besides GWT, FISH Oracle is built on the component library Smart GWT [51], a wrapper library for the SmartClient [52] JavaScript framework. This framework provides a large set of convenient software components (widgets), enabling the programmer to quickly implement a state-of-the-art user interface that is efficient, feature-rich and consistent. It should be noted that SmartClient also offers functionality for client-server communication. However, the server side library requires a commercial license conflicting with the open-source approach of FISH Oracle. Thus we used the client-server communication mechanisms provided by the GWT.

The large user community for GWT, comprising more than 1200 projects [53] (as of June 2011), and the fact that Google Inc. uses GWT as their central web development tool makes us confident that it will be maintained and improved in the remote future, so that applications depending on it can remain functional. For importing and exporting tabular data into and from FISH Oracle, the JExcel [54] and Java CSV [55] software libraries are used.

### Data visualization

For visualization of both segment and annotation data we used the *AnnotationSketch *[56] software library, a portable, fast and space-efficient annotation drawing solution that allows to display data from arbitrary sources, making it particularly suitable for an interactive web-based visualization tool. For efficiency reasons, *AnnotationSketch *was implemented in the C programming language. In order to access the drawing functions from FISH Oracle, an additional adapter layer between the C library and the Java virtual machine is required. As such an adapter, we used the Java Native Access library [57] (JNA) which allows to call C functions from Java programs. This enabled us to create Java counterparts for all components of the AnnotationSketch library, which were then used to implement the visualization functions in FISH Oracle. Our software architecture thus combines the advantage of having the time-critical image generation step implemented in a fast low level language (C) with the advantage of using a well-tested and widely used platform for dynamic web application development (Java). Figure S1 in the additional file 1 shows the data flow in FISH Oracle.

### Availability

FISH Oracle is available as a source code package via the FISH Oracle web site at http://www.zbh.uni-hamburg.de/fishoracle. It supports many POSIX conforming UNIX-like target platforms, for example Linux or Mac OS X.

On the web site we also offer additional documentation and a screencast video demonstrating the use of FISH Oracle.

## Authors' contributions

RS and SK conceived of the project. MM, SS and SK developed the software architecture. MM implemented the software and generated the results. SS contributed to the implementation of the data visualization. All authors wrote, read and approved the final manuscript.

## Supplementary Material

Additional file 1 **This PDF file contains additional tables comparing features of various other array CGH visualization software in detail, as well as an illustration depicting the data flow during user interaction with FISH Oracle**. Click here for file
