# Supplementary material for: FISH Oracle: a web server for flexible visualization of DNA copy number data in a genomic context
Source: J Clin Bioinforma. 2011 Jul 28;1:20. doi: 10.1186/2043-9113-1-20 (PMC3164613; doi:10.1186/2043-9113-1-20)
Supplement: Additional file 1 — This PDF file contains additional tables comparing features of various other array CGH visualization software in detail, as well as an illustration depicting the data flow during user interaction with FISH Oracle. [file 2043-9113-1-20-S1.PDF]

FISH Oracle: a web server for flexible visualization of DNA copy number data in a genomic context

Additional Data

Malte Mader, Ronald Simon, Sascha Steinbiss and Stefan Kurtz

July 26, 2011

Software feature	FISH Oracle	CGH-Explorer [19]	Caryoscope [20]	CGH-PRO [21]	CGHAnalyzer [22]	ChAR-MView [23]	Ideogram-Browser [24]	SnoopCGH [29]
Technology	GWT	Java	Java	Java	Java	Java	Java	Java
Interactive display	yes	yes	yes	yes	no	yes	yes	yes
Gene annotation visualization	yes	no	no	no	yes (not by default)	yes (user defined genomic feature)	yes	yes (load annotation file in GFF3 or EMBL)
Multiple genome support	yes	?	yes	?	?	yes	no	yes
Multiple microarray experiment comparison	yes	yes	no	yes	yes	yes	yes	yes
Multiple platform support	yes	?	yes	yes	yes	?	yes (emphasis on Affymetrix)	yes
Image export	yes (PNG, PDF, PS, SVG)	?	yes (PS, PDF, JPG, PNG)	yes (PNG)	?	yes	yes (JPG, SVG, PDF)	no
Data export	yes	?	no	?	?	yes	yes	no
Input formats	CSV	plain text, Microsoft Excel	CSV, GFF3	GenePix, Agilent, Imagene	standard text formats or database	CSV in PCL format	CNAT, CSV	CSV
Data/experiment types support	array CGH	array CGH, gene expressions data	array CGH, gene expressions data	array CGH	array CGH	array CGH, gene expression data	array CGH	array CGH, short read data
Integration of segmentation	no	yes (ACE)	no	aCGH [58], DNACopy	Algorithms from TIGR MeV [59]	ChARM [60]	no	Haar Wavelets [61], Smith-Waterman [62], RankCopy [63]
Integration of normalization	no	yes	yes	yes	?	?	no	yes
Integration of quality assessment	no	?	no	yes	?	?	no	no
Open source	yes	yes	yes	yes	?	yes	yes	yes
Availability	free	after registration	free	on E-Mail request	not available anymore	after registration	free	free
License	ISC	?	MIT	GPL	?	GPL	CC	GPL
Year of publication		2004	2004	2005	2005	2005	2006	2009

Table 1: Comparison of FISH Oracle with other desktop-based array CGH visualization software. Most software was published in the mid-2000s. “?” indicate that the information could not be obtained from the publication or publicly available documentation.

Software feature	FISH Oracle	SEURAT [27]	CHESS [28]	VAMP [25]	MD-SeeGH [26]	SIGMA^2 [30]
Technology	GWT	Java	Java	Java	C++/Windows	Java
Interactive display	yes	yes	yes	yes	yes	yes
Gene annotation visualization	yes	(chromosome view)	(very basic)	no	yes	no
Multiple genome support	yes	no	?	yes	yes	no (UCSC human)
Multiple microarray experiment comparison	yes	yes	yes	yes	yes	yes
Multiple platform support	yes	yes	yes	yes	yes	yes
Image export	yes (PNG, PDF, PS, SVG)	yes	no	?	yes	yes
Data export	yes	no	no	?	yes	yes
Input formats	CSV	CSV	CSV	CSV/XML	CSV	CSV
Data/experiment types support	array CGH	array CGH, gene expression data, SNP arrays	array CGH, gene expression data	array CGH, gene expression data, LOH, SNP arrays, CHIP-chip	array CGH, gene expression data, differential methylation, SNP arrays	array CGH, SNP arrays, CHIP-chip, gene expression data, SAGE
Integration of segmentation	no	no	yes (threshold, SW-ARRAY)	no	yes	yes
Integration of normalization	no	no	no	no	yes	no
Integration of quality assessment	no	no	no	no	yes	no
Open source	yes	yes	no	?	?	?
Availability	free	free	server unavailable	upon request	academic software license	free for academic use
License	ISC	GPL	?	?	?	?
Year of publication		2010	2009	2006	2008	2008

Table 2: Comparison of FISH Oracle with other desktop-based array CGH visualization software (continued). “?” indicate that the information could not be obtained from the publication or publicly available documentation.

Software feature	FISH Oracle	ArrayCyGHt [31]	arrayCGHbase [32]	CAPweb [33]	waviCGH [36]	ISACGH [35]	SIGMA [34]
Technology	GWT	PHP, Perl	PHP	PHP, Perl, Java (Applets)	YUI, Perl, Python	?	Java
Interactive display	yes	no	yes (chromosome view)	no	no	yes	yes
Gene annotation visualization	yes	no	yes (UCSC)	no	no	(via Ensembl)	no
Multiple genome support	yes	yes	yes	?	yes	yes, but fixed (human, mouse)	yes, but fixed (two human builds)
Multiple microarray experiment comparison	yes	no	yes	no	no	yes	?
Multiple platform support	yes	yes	yes	yes	yes	yes	yes
Image export	yes (PNG, PDF, PS, SVG)	?	yes (PNG, SVG)	?	yes	(via Ensembl)	yes
Data export	yes	?	MIAME compliant text files, BED	yes (to VAMP [25])	yes	?	yes
Input formats	CSV	GenePix, UCSC SPOT, Imagene, QuantArray	GenePix, Scanalyse, Imagene, Affymetrix (Chromosome Copy Number Tool)	GenePix, Imagene, Spot, MAIA	log-ratio, called data	CSV	? (own database)
User management	yes	no	yes	yes	no	?	yes
Data storage	MySQL	?	MySQL	MySQL	?	?	MySQL
Integration of segmentation	no	no	universal data Smoothing Algorithm, one breakpoint algorithm	GLAD [64]	HaarSeg [61], DNACopy, GLAD, Wavelets [65], HMM [58], BioHMM [66], CGHseg	?	no
Integration of normalization	no	yes	yes	yes	yes	?	no
Integration of quality assessment	no	yes	yes	no	no	?	no
Open source	yes	no	?	?	no	no	no
Availability	free	web-server	web-server (software available on request)	web-server (software available on request)	web-server	web server	free for research use
License	ISC	?	CC	?	?	?	?
Year of publication		2005	2005	2006	2010	2007	2006

Table 3: Comparison of FISH Oracle and other web-based applications. Except for WaviCGH, all applications were published in the mid-2000s. “?” indicate that the information could not be obtained from the publication or publicly available documentation.

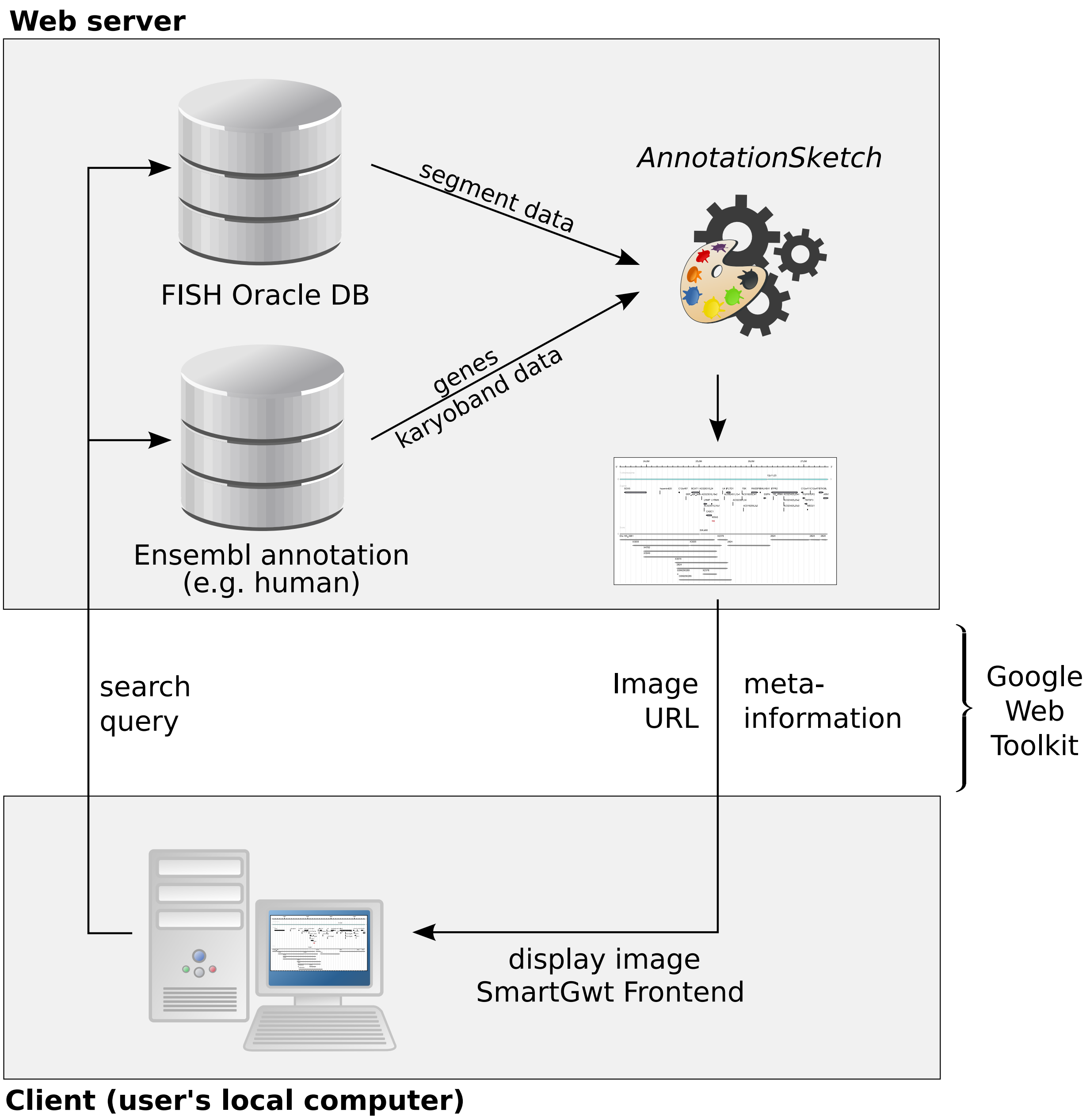

Figure 1: Data flow during user interaction with the FISH Oracle application. A search query and additional query options are sent from the browser on the user's local machine to the server. This information is processed on the server by combining information from the Ensembl and FISH Oracle database, finally generating an image using the *AnnotationSketch* software. In addition to the URL of the final image, additional meta-information such as pixel positions of genomic elements or displayed range information is sent back to the user's browser which displays the retrieved information.
